# Ciliated epithelia are key elements in the recruitment of bacterial partners in the squid-vibrio symbiosis

**DOI:** 10.3389/fcell.2022.974213

**Published:** 2022-10-20

**Authors:** Katrina A. Gundlach, Janna Nawroth, Eva Kanso, Farzana Nasrin, Edward G. Ruby, Margaret McFall-Ngai

**Affiliations:** ^1^ Kewalo Marine Laboratory, University of Hawaiʻi at Mānoa, Honolulu, HI, United States; ^2^ Helmholtz Pioneer Campus, Helmholtz Zentrum München, Neuherberg, Germany; ^3^ Department of Aerospace and Mechanical Engineering, University of Southern California, Los Angeles, CA, United States; ^4^ Department of Mathematics, University of Hawaiʻi at Mānoa, Honolulu, HI, United States

**Keywords:** ciliated fields, symbiont harvesting, *Euprymna scolopes*, *Vibrio fischeri*, light organ, ciliary beat frequency

## Abstract

The Hawaiian bobtail squid, *Euprymna scolopes*, harvests its luminous symbiont, *Vibrio fischeri*, from the surrounding seawater within hours of hatching. During embryogenesis, the host animal develops a nascent light organ with ciliated fields on each lateral surface. We hypothesized that these fields function to increase the efficiency of symbiont colonization of host tissues. Within minutes of hatching from the egg, the host’s ciliated fields shed copious amounts of mucus in a non-specific response to bacterial surface molecules, specifically peptidoglycan (PGN), from the bacterioplankton in the surrounding seawater. Experimental manipulation of the system provided evidence that nitric oxide in the mucus drives an increase in ciliary beat frequency (CBF), and exposure to even small numbers of *V. fischeri* cells for short periods resulted in an additional increase in CBF. These results indicate that the light-organ ciliated fields respond specifically, sensitively, and rapidly, to the presence of nonspecific PGN as well as symbiont cells in the ambient seawater. Notably, the study provides the first evidence that this induction of an increase in CBF occurs as part of a thus far undiscovered initial phase in colonization of the squid host by its symbiont, i.e., host recognition of *V. fischeri* cues in the environment within minutes. Using a biophysics-based mathematical analysis, we showed that this rapid induction of increased CBF, while accelerating bacterial advection, is unlikely to be signaled by *V. fischeri* cells interacting directly with the organ surface. These overall changes in CBF were shown to significantly impact the efficiency of *V. fischeri* colonization of the host organ. Further, once *V. fischeri* has fully colonized the host tissues, i.e., about 12–24 h after initial host-symbiont interactions, the symbionts drove an attenuation of mucus shedding from the ciliated fields, concomitant with an attenuation of the CBF. Taken together, these findings offer a window into the very first interactions of ciliated surfaces with their coevolved microbial partners.

## Introduction

Many animals form symbioses with beneficial microbes through horizontal transmission, where each generation of animals must initiate their symbiotic associations anew with environmentally acquired symbionts (Bright and Bulgheresi, 2010). Marine environments typically have a background of millions of microbes in the surrounding seawater, with symbionts often representing only a small fraction of the pico- and nanoplankton populations. As such, horizontal transmission of symbionts in these habitats presents a challenge for the efficient colonization of nascent symbiotic tissues.

Ciliated epithelial surfaces are widespread among marine animals, but the extent to which they promote interactions with environmental symbionts during horizontal transmission is poorly understood. Motile cilia are evolutionary conserved organelles found throughout the animal kingdom (Satir and Christensen, 2007). As projections from the cell surface, motile cilia can generate flow-fields that are often used by aquatic animals, in concert with shed mucus, for feeding and/or the clearance of fouling particles (Brown and Bythell, 2005; Shapiro et al., 2014; Marinković et al., 2020). In a recent study of the binary symbiotic association between the bobtail squid *E. scolopes* and its luminous bacterial partner *V. fischeri*, ciliary-mucus flow fields that are associated with nascent symbiotic tissues were demonstrated to have characteristics that promote enrichment of symbiont-sized particles in regions near the sites of colonization (Nawroth et al., 2017). Because this experimental system allows the direct observation of the initiation of the symbiosis, the findings of Nawroth et al. (2017) suggested a rich landscape for in-depth characterization of the role of cilia in the initiation of a horizontally transmitted animal symbiosis.

**FIGURE 1:**
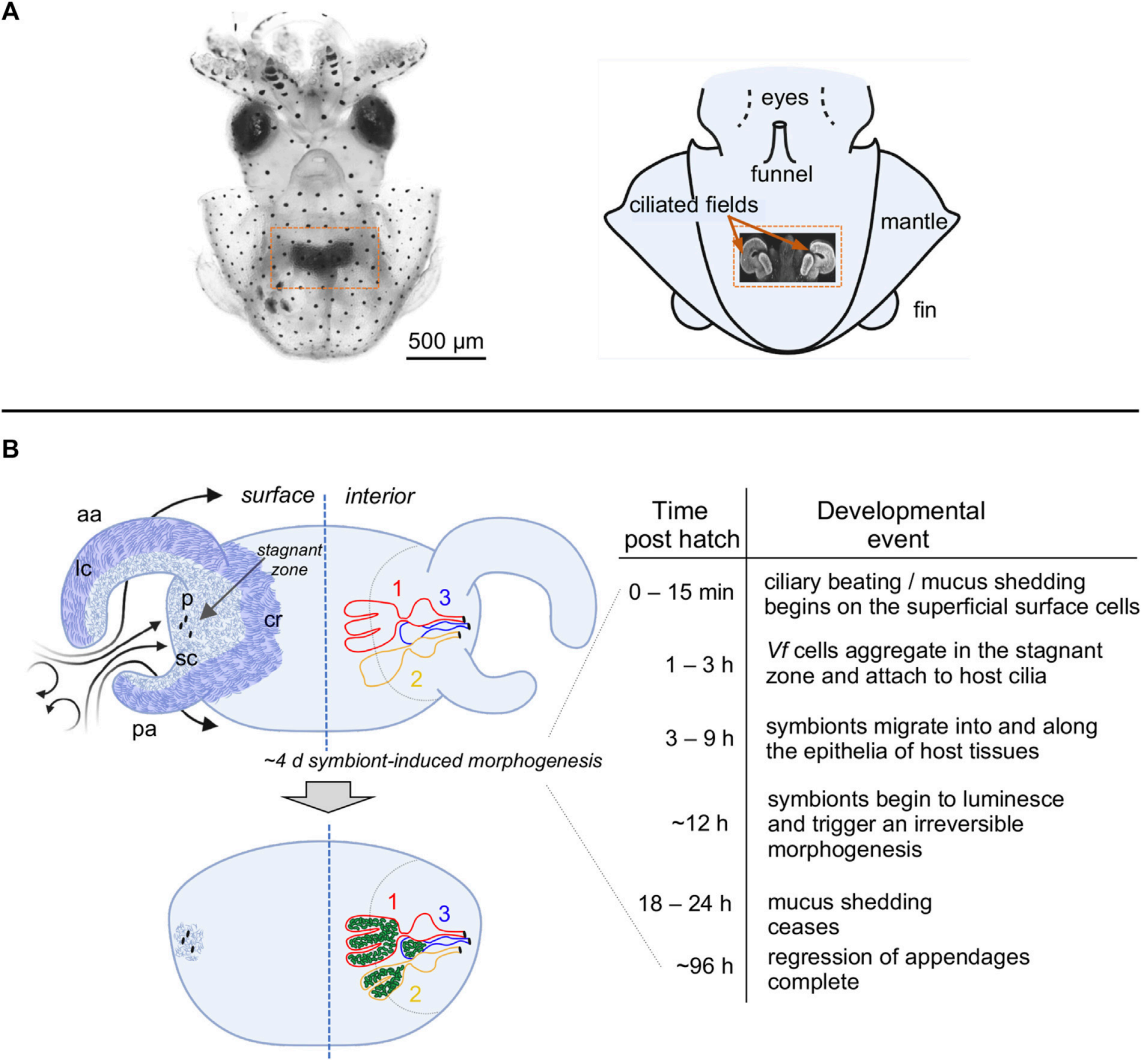
The light organ of *Euprymna scolopes*, and relevant early post-hatch events in the establishment of symbiosis. **(A)** The position of the organ in the body cavity. Left, a light micrograph of a juvenile *E. scolopes*, showing the location of ink-sac-associated light organ within the body cavity (orange box). Right, a diagram of a hatchling squid with a portion of the mantle removed to reveal a confocal micrograph (inset) of the internal light organ with its lateral ciliated fields (orange arrows). **(B)** Early development of the light organ under natural conditions post symbiont colonization (left) and the timeline of the associated events (right). Upper left—at hatching, each exterior lateral surface is covered with two distinct populations of cilia: (i) longer metachronally-beating cilia (lc; darker blue areas) along the outer regions of the anterior (ap) and posterior (pa) appendages, and along the medial ciliated ridge (cr); and, (ii) shorter randomly beating cilia (sc) (lighter blue areas), which cover the inner surfaces of the appendages, and surround the pores (p) into which the symbionts migrate. Following recruitment to a stagnation zone above the pores, *V. fischeri* cells will migrate into the pores and down a migration path into three blind-ended crypts (1, 2, 3) on each side of the organ, where they will proliferate and populate these spaces. Lower left—by ∼4d, colonizing symbionts (green) induce a developmental program that results in a gradual loss of the superficial ciliated fields.

The *Euprymna scolopes-Vibrio fischeri* association has been studied for over three decades to discover and understand host-bacteria symbiotic interactions (for reviews see, Nyholm and McFall-Ngai 2021; Visick et al., 2021). During embryogenesis, the squid develops a nascent symbiotic structure, or light organ, in the center of the body cavity. The organ bears a pair of ciliated fields, one on each lateral face of the nascent structure (Figure 1). Each ciliated field comprises two appendages and a region associated with the organ surface, where it surrounds three pores. Shortly after hatching, the host sheds mucus from these superficial ciliated fields. With each ventilatory movement of the squid’s mantle, environmental seawater containing the bacterioplankton is brought into the body cavity. Nawroth et al. (2017) showed that inanimate particles of a certain size, as well as symbionts, are enriched in regions above the pores of the organ, where the latter attach to host cilia and form small aggregates. After some residence time, these cells migrate toward and into the pores of the light organ. Once through a pore, the *V. fischeri* cells follow a migration path into crypts, where the symbionts grow out and colonize for the remainder of the lifetime of the animal. From the crypt spaces, the symbionts signal the attenuation of mucus production from the ciliated fields within 24 h, as well as the eventual loss of these fields through an extensive symbiont-induced apoptosis of the epithelial cells (Montgomery and McFall-Ngai, 1994).

Here, to study the role of the superficial ciliary fields in potentiating symbiont colonization, we developed assays to measure ciliary beat frequency (CBF) across the light organ using high-speed videography coupled with MATLAB (MathWorks, Portola Valley, CA, United States) analysis, which provided high temporal and spatial resolution of the CBF measurements. We investigated: 1) the impact of exposure to the natural environment at hatching on the efficiency of the ciliated fields in the harvesting of the symbiotic partner; 2) the biochemical mechanisms underlying these influences; and, 3) the effects of symbiont exposure and light-organ colonization on the behavior of these tissues. Our findings provide insights into the mechanisms by which ciliated surfaces may foster the interactions of animal hosts with their specific microbiome.

## Materials and methods

### General

Adult *E. scolopes* were collected from Maunalua Bay, Oahu, and maintained in flow-through offshore seawater (OSW) at Kewalo Marine Laboratory. Unless otherwise specified, all experiments were conducted in OSW, either unfiltered or filtered (FOSW; 0.22-μm pore size). For all experiments in which CBF was measured, juvenile squid were anesthetized (2% ethanol v/v) and the light organs were dissected out of the mantle cavity. For CBF analyses, images of light organs were viewed on a Zeiss Axioskop 2, and recorded by high-speed videography (Sony FDR-AX700, image size 1,080 × 1,920 pixels). The *V. fischeri* inocula were prepared by growing the bacteria in LBS medium overnight as previously described (Naughton and Mandel, 2012); several mutant derivatives of a native light-organ isolate were used in the experiments (Table 1). All chemicals were purchased from Sigma-Aldrich (St. Louis, MO, United States), unless otherwise noted. Successful colonization of animals was determined using emission of symbiont luminescence as detected by a luminometer (Turner Designs, Inc., Sunnyvale, CA, United States). In curing experiments, colonized animals were treated with 10 μg ml^−1^ chloramphenicol at 12 h post-inoculation and maintained under antibiotic pressure for the 24 h (Doino and McFall-Ngai, 1995). Curing was confirmed both by the loss of luminescence and by plating the organs for colony-forming units (CFUs).

**TABLE 1:** Derivatives of *Vibrio fischeri* strain ES114 used in this study.

Strain	Genetic	Characteristics	Reference
ES114	parental strain	Wild type	Boettcher and Ruby (1990)
JRM200	Cm^R^ insertion in Tn*7* site	Chloramphenicol-resistant	McCann et al. (2003)
DM73	spontaneous mutant	Hyperflagellated swarmer	Millikan and Ruby (2002)
ES114 *flrA*	*VF_1856::TnErm*	Unflagellated, and non-motile	Aschtgen et al. (2016)
ES114 *motB1*	*VF_0715::TnErm*	Flagellated, but non-motile	Aschtgen et al. (2016)
ES114 *pilT*	*VF_0431::TnErm*	Missing PilT pilus	C. Bongrand (unpubl.)
ES114 *waaL*	*VF_0151::TnErm*	Missing LPS	O-antigen Post et al. (2012)

### Fluorescence microscopy

Fluorescent staining and imaging were carried out as previously described (Davidson et al., 2004). The following fluorescent dyes were used: 4-amino-5-methylamino-2′,7′-difluorescein diacetate (DAF-FM DA) from Thermo Fisher Scientific (Waltham, MA, United States) was used to detect presence of NO; wheat-germ agglutinin WGA Alexa Flour 633 (Thermo Fisher) was used to stain mucus; and, Cell Tracker Orange (Thermo Fisher) was used to label host tissues. The confocal fluorescent microscopy was performed using a Zeiss LSM 710 confocal microscopy (Carl Zeiss AG, Jena, Germany), stationed at the University of Hawai’i, Mānoa (UHM), Kewalo Marine Laboratory.

### Exploring the role of peptidoglycan-induced mucus shedding on ciliary beat frequency

To determine the influence of post-hatch mucus shedding on CBF, hatchling squid were transferred to filter-sterilized (0.22-μm pore size) artificial seawater (ASW) within 5 min of hatching to control their exposure to environmental peptidoglycan (PGN), PGN was from Invitrogen (San Diego, CA, United States). Cohorts of these squid were then exposed to lysozyme-digested PGN for 2 h (200 μg ml^−1^) in ASW to mimic natural exposure to PGN in OSW or ASW alone (Nyholm et al., 2000); lysozyme was obtained from Thermo Fisher Scientific (Waltham, MA, United States). The levels of nitric oxide (NO) in host-shed mucus were experimentally reduced by adding either the nitric-oxide synthase (NOS) inhibitor *S*-methyl-L-thiocitrulline (SMTC), or the NO scavenger rutin hydrate (RH); animals were preincubated with PGN alone in ASW for 30 min, and then the inhibitor or scavenger was added at a concentration of 100 μM, respectively, for another 1.5 h (Davidson et al., 2004; Wang et al., 2010). In contrast, the ambient NO concentration was increased by adding the NO donor diethylamine nonoate (DEA-NONOate), which was reconstituted in 10 mM NaOH just prior to addition. Animals were treated with DEA-NONOate in ASW at a concentration of 1 mM for 10 min (Wang et al., 2010). Alternatively, animals were treated with L-arginine at a concentration of 10 mM for 30 and 60 min to increase ambient NO through the NOS reaction (Jiao et al., 2010).

### Colonization efficiency experiments

To determine whether the observed increase in CBF that occurs with mucus shedding has a significant effect on colonization of the light organ, as juvenile squid emerged from the egg they were immediately transferred to ASW to prevent the exposure to environmental PGN from inducing mucus shedding. Then, the animals were exposed to one of two conditions; either ASW containing lysozyme-treated PGN (200 μg ml^−1^), or lysozyme alone. Then they were presented with one of three inoculum concentrations: 500, 800, or 1,200 CFU ml^−1^ for 3 h; these conditions typically result in <100% colonization (McCann et al., 2003). After 3 h, the animals were washed in fresh ASW and kept in individual vials with water changes every 12 h. Luminescence, as an indication of colonization, was recorded at 24 h and 48 h.

### High-speed video recording

Light organs were dissected from animals anesthetized in 2% (vol/vol) ethanol in ASW, OSW, or FOSW, depending on the experiment. The organs were then placed in a depression slide containing the treatment solution associated with a given experiment. Preparations were viewed using light microscopy with differential interference contrast (DIC). The motion of the surface cilia on the light organs was recorded using a high-speed camcorder (Sony FDR-AX700, image size 1,080 × 1,920 pixels), recording at either 240 or 960 frames per second (fps).

### Analysis and statistics of ciliary beat frequencies

Ciliary beat frequency (CBF) was measured by applying Fourier spectral analysis to high-speed DIC video recordings of the ciliated light organ, as previously described (Benam et al., 2016; Nawroth et al., 2017). A MATLAB-based algorithm was used to detect regions of cilia movement. The field of view was divided into windows of 30 × 30 pixel, where the CBF of each window was calculated individually. The gray value of each pixel over time was analyzed using fast Fourier transform (FFT). The dominant frequency of the power spectra for all pixels in that window corresponds to the CBF. A heatmap was generated which compiled all the CBFs of the windows within the field of view.

### Statistical analysis

Data were analyzed using GraphPad Prism software, version 8.4.2 (GraphPad Software, Inc., La Jolla, CA, United States). Data from each experimental condition passed a D’Agostino and Pearson omnibus test of normality. Data were then compared using a one-way analysis of variance (ANOVA) and a Tukey’s *post hoc* test (*α* = 0.05). Data are expressed as means ± SEM. A value of *p* < 0.05 was considered statistically significant. For analysis of the effects of PGN-induced mucus shedding on colonization, a paired sample *t -*test was performed using the null and the alternative hypothesis corresponding to the two conditions being either not different or statistically different, respectively.

## Results

### Exposure to natural seawater caused a post-hatch increase in ciliary beat frequency

**FIGURE 2:**
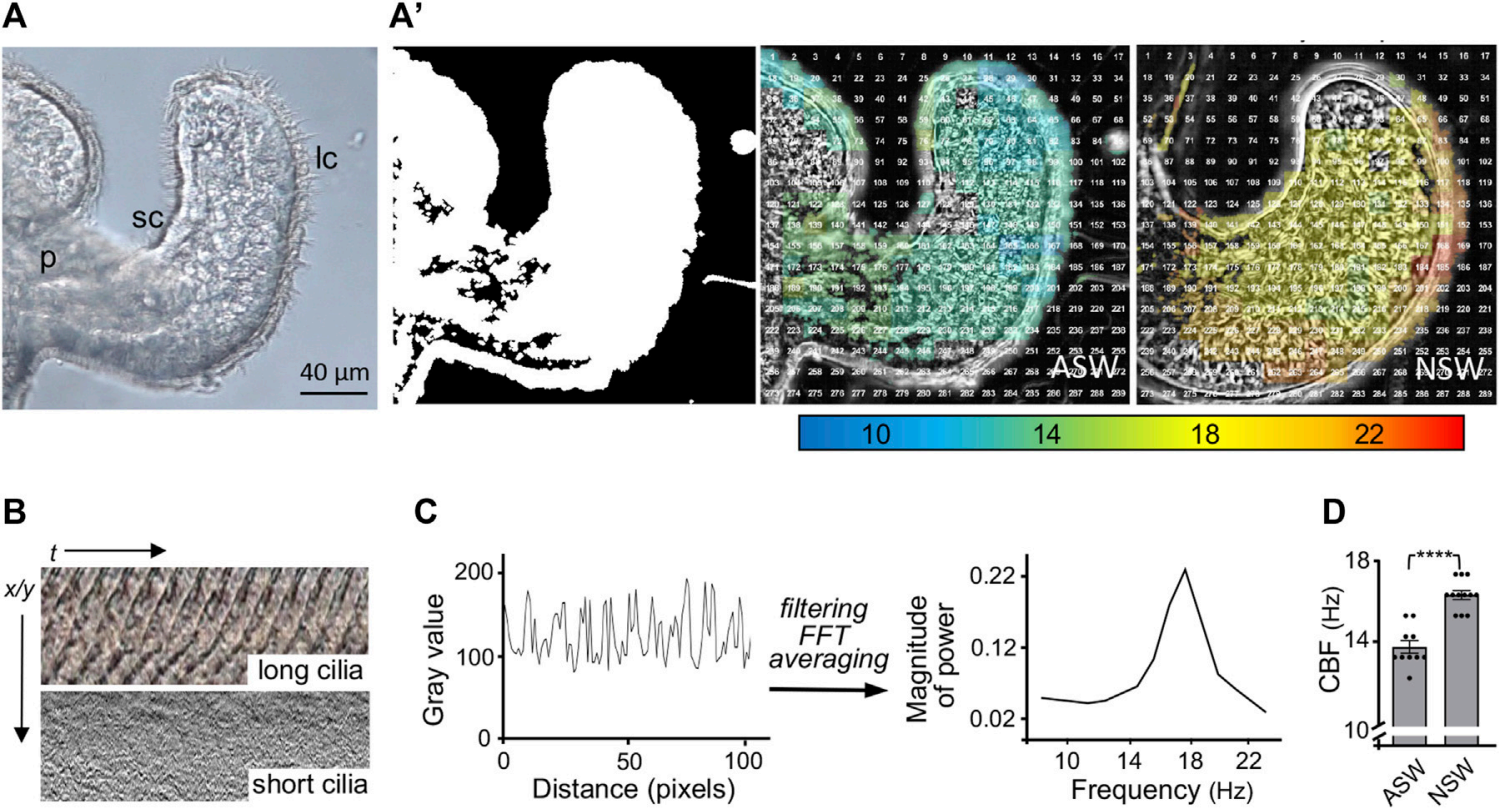
Analyses of the ciliated field that potentiates host colonization by the bacterial symbiont. **(A)** A still image from a high-speed video (×40, DIC, 1,000 frames/sec) of one half of the hatchling squid light organ, showing the long (lc) and short (sc) cilia; p, pore. **(A′)** Left, a MATLAB algorithm detects areas of motion (white); middle and right, speed of ciliary beat, calculated using fast Fourier transform (FFT) of each window, at 30 min post hatching of the host animal into either artificial (ASW) or natural (NSW) seawater. **(B)** Kymographs showing the changes in gray values over time of the ciliary beat in the long (metachronally beating) and short (randomly beating) cilia, respectively; *t*, time; *x/y* = pixel location. **(C)** Periodic changes in gray value over distance (pixels), which represents time corresponding to the beat pattern of the metachronally beating, long cilia. Filtering of high and low frequency noise and artifacts, followed by FFT and averaging to extract a spectrum of frequencies (Hz) at this location. **(D)** Derived ciliary beat frequency (CBF) of animals exposed for 30 min post hatching to either ASW or NSW; CBF (Hz), ciliary beat frequency in Hertz; (*n* = 10); ****, *p* < 0.0001.

We developed methods to characterize the CBF patterns across excised light organs by recording high-speed videos and analyzing the detected motion in these videos using a MATLAB algorithm (Figure 2). The first analyses with these methods determined that newly hatched animals exposed for 30 min to natural seawater, which contains bacteria and their exported products (like PGN), had a significantly higher CBF across their light organ than animals hatched into artificial seawater (Figure 2D).

### Nitric oxide vesicles in host-shed mucus increased ciliary beat frequency, thereby influencing colonization efficiency

**FIGURE 3:**
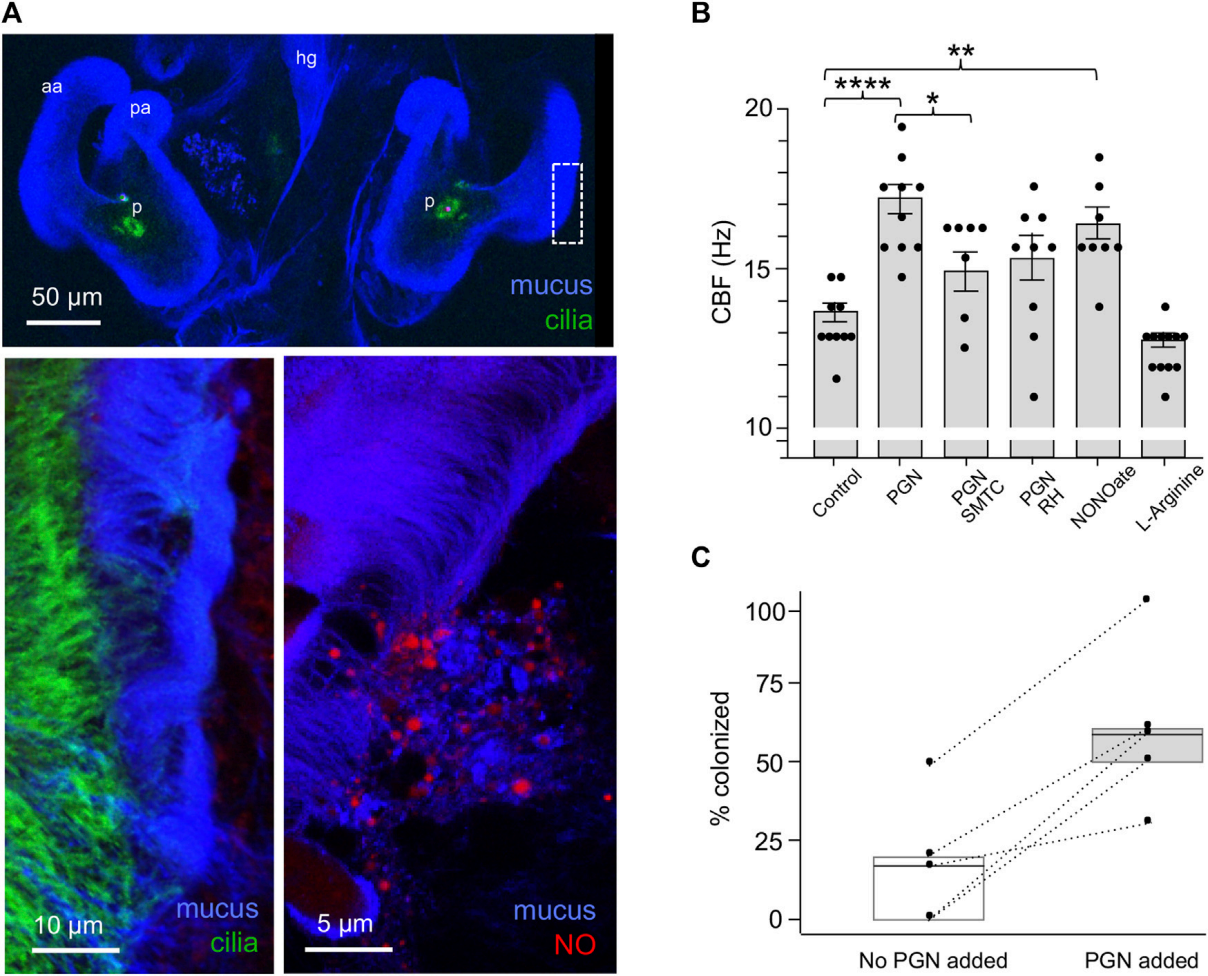
The influence on CBF of peptidoglycan (PGN)-induced mucus shedding in the hatchling light organ. **(A)** Confocal images of relevant post-hatch events. Top, within minutes, PGN shed from environmental bacteria has induced a non-specific release of copious mucus from the superficial ciliated fields on either side of the organ; in this image the labeling of the cilia is obscured by the mucus, except at the pores (p). aa, anterior appendage; hg, hindgut; pa, posterior appendage. Lower left—region of dashed box in top image rotated around its axes to show the mucus as it typically resides above the ciliated field. Lower right—NO (red) occurs in vesicles in the mucus. **(B)** Responses of CBF to the presence of PGN, and manipulation of the NO cue present in the mucus. (See Materials and Methods for conditions of exposure.) *p < 0.05; **p < 0.01; ***p < 0.001; ****p > 0.0001; ns, not significant. **(C)** The effect of PGN-induced mucus shedding on host colonization. Animals were exposed to 500–800 *V. fischeri*/ml of seawater with either no PGN or 200 µg PGN/ml added for 2 h, and the luminescence was measured after 24 h; all animals that subsequently emitted luminescent were considered colonized; five replicates were made from different clutches of host eggs; each replicate had 10–14 animals/condition. The summary statistics of these two experimental conditions are a mean of (i) 17 (±19.7) without added PGN, and (ii) 60 (±25.5) with added PGN. In a paired plot of these two conditions, the dashed lines between points connect the two replicates, and the boxes are the interquartile ranges for each condition. Both the summary and pairing statistics suggest that further analysis would be informative. Nevertheless, to determine the effect of PGN-induced mucus shedding, we used a paired-sample *t*-test, with the null and the alternative hypotheses corresponding to the two conditions being either not different or statistically different, respectively. The *t-* test’s *p*-value (0.0058) indicates that the two conditions have significantly different outcomes.

Earlier studies of the system had shown that PGN induces mucus shedding from the ciliated field (Nyholm and McFall-Ngai, 2004). In addition, vesicles containing nitric oxide (NO) occur in this mucus, and this NO diffuses into the surrounding milieu (Davidson et al., 2004; Figure 3A). Nevertheless, no putative function for these phenotypes had been defined. Here we found that PGN-induced mucus shedding (Nyholm et al., 2002) results in a significant increase in CBF (Figure 3B). It should be noted that the mucus occurs as discontinuous patches above the ciliated field (Figure 3A); it is in those areas not covered by mucus that the cilia-driven flows can recruit *V. fischeri* cells from the overlying seawater. In addition, because where mucus is present it occurs as a layer above the cilia, it does not physically affect the CBF. Because NO is known to influence CBF (Jiao et al., 2011), we manipulated NO levels in the mucus, and found that inhibitors of NO significantly compromised the normal increase in CBF, while addition of DEA-NONOate, a potentiator of ambient NO levels, significantly increased CBF. In contrast, the addition of L-arginine, a substrate for NOS, did not increase CBF (Figure 3B). Taken together these data implicate the NO pathway as the regulatory system responsible for controlling the CBF increase as a response to the NO present after PGN-induced mucus shedding.

To investigate the implications of CBF on symbiosis initiation, we measured colonization success of animals inoculated with low *V. fischeri* concentrations in the absence or presence of PGN-induced mucus shedding (Figure 3C). To control for the differences in the colonization efficiency of animals from different clutches, we compared responses to the two conditions using animals from the same clutch (*n* = 10–14 animals/condition over five trials). In all five trials, the absence of PGN-induced mucus shedding reduced colonization by 24 h compared to colonization in the presence of PGN-induced mucus shedding. Thus, the PGN-induced increase in CBF may well underlie the enhanced the efficiency of colonization by *V. fischeri*.

### Addition of *V. fischeri* cells in the background of abundant bacterioplankton increased ciliary beat frequency

**FIGURE 4:**
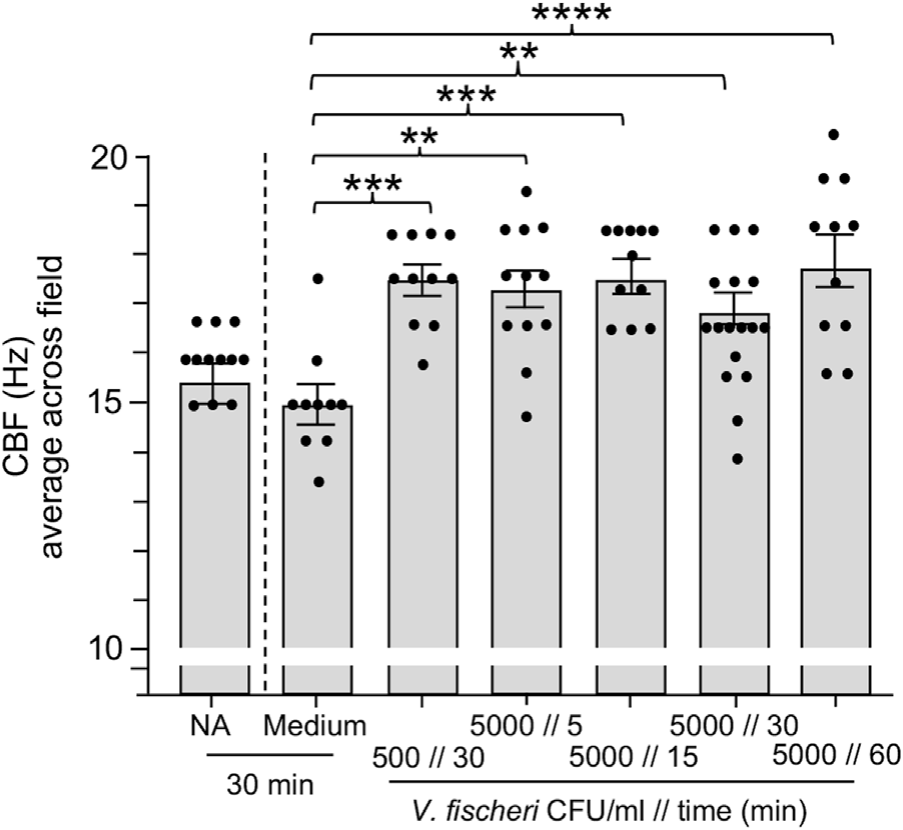
Ciliary beat frequency (CBF) measurements of hatchling light organs show exquisite sensitivity to *Vibrio fischeri*. Animals were incubated with various concentrations of *V. fischeri* (*Vf*) in natural seawater containing ∼10^6^ CFU/ml of other marine bacteria. As controls, animals were also exposed to only natural seawater with no additions (NA) or to addition of the same volume of cell-free 0.22 µm-filtered spent LBS-medium as presented with *Vf* cells (Medium). A one-way ANOVA and Tukey’s *post hoc* test were used (F_6, 79_ = 6.251, *p* < 0.0001). Animals (*n*) per treatment: NA, *n* = 12; medium control, *n* = 10; medium with 500 CFU/ml for 30 min, *n* = 13; 5,000 CFU/ml 5 min, *n* = 12; 5000 CFU/ml 15 min, *n* = 11; 5,000 CFU/ml 30 min, *n* = 17; 5,000 CFU/ml, 60 min, *n* = 11.

In habitats supporting the squid-vibrio symbiosis, *V. fischeri* cells typically represent <0.1% of the bacteria in the plankton, i.e., <10^3^ cells·ml^−1^ in a background of ∼10^6^ cells·ml^−1^ of other species. In the results described above, we showed that exposure to the bacteria present in OSW caused an increase in CBF even in the absence of *V. fischeri*. Subsequent experiments revealed that the host ciliated fields sensed the addition of *V. fischeri* even within this large background of other, non-specific cells, i.e., a significant increase in CBF was observed when animals were exposed to *V. fischeri* cells for as short a period as 15 min (Figure 4). The addition of a small amount of the culture medium with the inoculum was not responsible for this response; i.e., addition of the medium alone produced no response. Taken together, these data provide evidence that this squid is exquisitely sensitive to the presence of its natural bacterial partner, even with the abundant background “noise” of the normal bacterioplankton.

We sought to connect this increase in CBF to the corresponding increase in the velocity of cilia-driven flows, and the effect of these flows on the transport of bacteria across the ciliated surface (Kanso et al., 2021). In a previous study, we found that cilia-generated vortices actively filter bacteria-sized particles from the ambient fluid in the squid mantle cavity into a ciliated “stagnation zone” at the organ’s surface just above the pores (Figure 1B). To assess the nature of these ciliated flows, we first computed the Reynolds number Re, defined as *Re = ρUL/η*, where *ρ* and *η* are the water density and viscosity, *L* is the length scale of the ciliated appendages, and *U* is the speed of the cilia-generated currents. For *ρ = 1 g cm* ^*−3*^ and *η = 8.9 · 10* ^*–3*^ *g cm* ^*−1*^ *· s* ^*−1*^ at 25°C and, considering *L = 200 μm* (*0.02 cm*) and *U = 500 μm s* ^*−1*^ (*0.05 cm s* ^*−1*^), we calculated Re ≈ 10^–4^ (Nawroth et al., 2017). At such a low Re, viscosity is dominant, and the fluid motion is governed by the incompressible Stokes flow model. We then estimated the amount of fluid that the cilia-generated microcurrents allow the light organ to process across the surface per unit time. We used a flow speed *U = 500 μm s* ^*−1*^ and we estimated the area *A* of the stagnation zone at each of the two sides of the organ. The stagnation zone can be approximated as a disc of radius *r = 50 μm and area A = πr* ^*2*^. The cilia-generated microcurrents filter ambient fluid at a volume flow rate *Q = U · A = 0.8 μm* ^*3*^ *s* ^*−1*^ on each side of the light organ. By fluid continuity and linearity of the Stokes flow regime, the total volume flow rate per light organ is *2Q*. Given that the cavity volume of a nascent squid is about *V = 1* *μl = 1*,*000 μm* ^*3*^, the amount of time it would take to process an equivalent volume of fluid by the light organ is *V/2Q =* 625 s. Thus, the entire volume of the body cavity might be processed in about 10 min, or alternatively, at a processing rate of about 0.1 μl/min. The cavity’s fluid content is gradually refreshed through respiration (Nawroth et al., 2017). It is reasonable to think that, given the recirculatory nature of the cilia-generated microcurrents, a given fluid “parcel” might be processed repeatedly, while missing bacterial cells that are not aligned with the fluid streamlines that reach the light organ boundary. Perturbations to these streamlines by spontaneous movements of the ciliated appendages as well as the bulk transport of the fluid inside the cavity by the animal’s respiration. These perturbations likely help decrease the probability that any volume of seawater is processed repeatedly. However, the estimated sampling rate should be viewed as an upper limit. The processing time is likely to take much longer.

Does the empirically observed increase in CBF significantly affect the fluid processing time? By the linearity of Stokes flow, it is reasonable to assume that an increase in the CBF corresponds to a proportional increase in the speed of the cilia-generated microcurrents. For example, an increase in CBF from 12.5 to 15 Hz means a 20% increase in speed *U* and volume flow rate *Q*, whereas an increase from 12.5 to 17.5 Hz corresponds to a 40% increase in *U* and *Q*. In turn, a 20% increase in *Q* leads to a 17% decrease in the fluid processing time, and a 40% increase in *Q* leads to a 29% decrease in processing time. These effects can be significant; for example, if it takes 10 min to encounter a sufficient number of *V. fischeri* cells in the stagnation zone at the base CBF, potential exposure to these bacteria would take only 7 min with a 40% increase in CBF. Realistically, even this rate appears too slow to account for the robust signaling detected after only 15 min of exposure to an inoculum of 5 cells/μl (Figure 4). Thus, it appears likely that some effect beyond the passage of *V. fischeri* cells across this surface zone must play a role in driving an increase in CBF.

**FIGURE 5:**
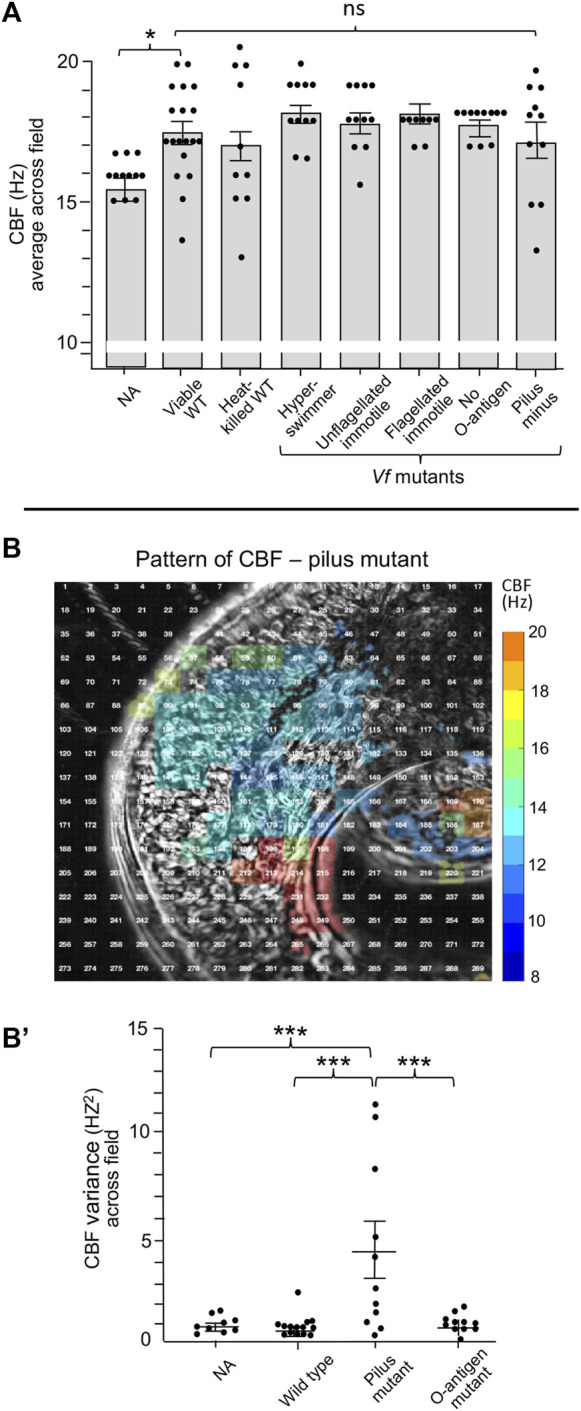
Ciliary beat frequency (CBF) measurements of hatchling light organs show no effect of symbiont viability or certain surface features or behaviors. **(A)** Conditions and features that show no difference in CBF and pattern of CBF in comparison with exposure to viable wild-type *V. fischeri*. In all conditions, animals were exposed to 5,000 *V. fischeri* CFU/ml for 30 min and then CBF was measured. For statistical analyses one-way ANOVA and Dunnett’s multiple comparisons test were used (F_5, 67_ = 4.86, *p* = 0.335). Animals (*n*) per treatment: viable, *n* = 19; heat-killed, *n* = 10; hyperswimmer, *n* = 11; unflagellated immotile, *n* = 11; flagellated immotile, *n* = 8; no O-antigen, *n* = 11. For the pilus mutant, a one-way ANOVA and Tukey’s *post hoc* test were also used (F_3,47_ = 3.428, *p* = 0.0245), *n* = 11; **(B,B′)** While exposure to the pilus mutant showed no overall difference in CBF **(B)**, an example of a single animal; see Figure 2 for the heat-map patterns for wild-type *V. fischeri*, the variance of CBF measurements between individual light organs was significantly higher in animals exposed to the mutant **(B′)**. Significantly different values are indicated as: *p < 0.05; **p < 0.01; ***p < 0.001; ****p < 0.0001; ns, not significant.

We then attempted to define features of *V. fischeri* cells that might be responsible for this sensitivity by deleting them. However, neither heat-killed *V. fischeri* cells nor mutant cells defective in flagella structure/function, endotoxin O-antigen or pilus production showed a significant difference in CBF from that of untreated or wild-type cells (Figure 5A). Interestingly, while exposure to the pilus mutant resulted in a much higher variance in CBF across the ciliated field (Figures 5B,B′), this phenotype did not influence colonization efficiency of the pilus mutant.

### Within 24 h of organ colonization by *V. fischeri*, ciliary beat frequency was attenuated

**FIGURE 6:**
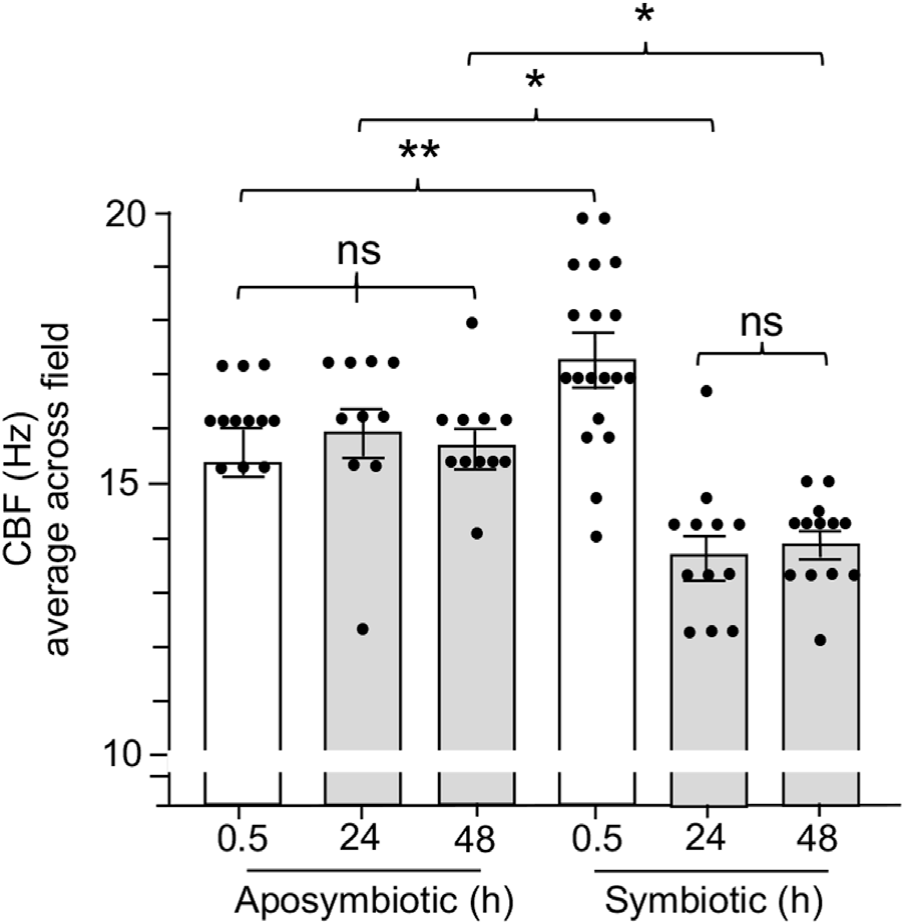
The effect of light organ colonization on CBF. Animals were inoculated with 5,000 CFU *Vibrio fischeri* per ml of NSW for symbiotic treatment, or only natural seawater (NSW) for aposymbiotic treatment. For treatments longer than 0.5 h, animals were rinsed twice in NSW after 3 h of exposure to the inoculum of *V. fischeri* and maintained in NSW for the remainder of experiment. CBF measurements were recorded at 0.5 h, 24 h, and 48 h. A one-way ANOVA and Tukey’s *post hoc* test were applied to the results (F_5, 72_ = 14.45, *p* < 0.0001). Animals (*n*) per treatment: 0.5 h aposymbiotic, *n* = 12; 24 h aposymbiotic, *n* = 10; 48 h aposymbiotic, *n* = 11; 0.5 h symbiotic, *n* = 19; 24 h symbiotic, *n* = 12; 48 h symbiotic, *n* = 13. Significantly different values are indicated as such: *p < 0.05; **p < 0.01; ns, not significant.

Previous work on the squid-vibrio system has demonstrated that colonization of the juvenile organ causes cessation of mucus shedding from the superficial ciliated fields by 24 h (Nyholm and McFall-Ngai, 2021). Thus, we sought to determine whether CBF was also influenced by colonization. Such experiments found that CBF is attenuated concomitant with the loss of mucus shedding. The CBF of symbiotic light organs after 24 h and 48 h is significantly slower when compared to aposymbiotic animals at the same times (Figure 6). These data further reinforce the link between the presence of mucus and elevated CBF in the epithelia that potentiate symbiont colonization.

## Discussion

The squid-vibrio model is a naturally occurring two-partner association that has provided the rare opportunity to document symbiont selection and colonization under both natural and experimental conditions. This present contribution explored the characters of juvenile-specific ciliated epithelia that potentiate efficient recruitment of the specific symbiont in the minutes to hours following the hatching of the juvenile host.

The data here provide evidence that evolution has selected for the development of features during embryogenesis that mediate immediate responses of the light-organ ciliated fields once the juvenile squid hatches into the environment (Montgomery and McFall-Ngai, 1993). Specifically, within minutes, host exposure to PGN derivatives released by the bacterioplankton causes the shedding of NO-containing mucus from the ciliated fields (Nyholm et al., 2000; Davidson et al., 2004). Our data show that the resulting increase in CBF enhances the colonization process. The CBF increase driven by the shedding of NO-containing mucus is a widely reported phenomenon in the ciliated surfaces of other animals (for review see Fang and Vasquez-Torres, 2019), but whether this behavior is critical for initial colonization by the normal microbiota has not been studied in other systems to our knowledge. However, resistance to NO is a well documented trait in both pathogens and the normal microbiota as a requirement for successful colonization of host cell surfaces, and the modulation of NO levels is known to be critical for homeostasis of host-microbiota interactions (Freund et al., 2018; Kuek and Lee, 2020; Porrini et al., 2020).

Our findings provide exciting new windows into the colonization process of the squid-vibrio association. Most unexpected was the discovery of an immediate increase in CBF in response to the presence of *V. fischeri*, revealing an exquisite sensitivity to the symbiont even within the rich background of other bacteria in the environment. The timing of these early events suggests also that the changes in CBF in response to both the presence of *V. fischeri* and to environmental PGN occur at about the same time following hatching, i.e., within minutes, suggesting that, under natural conditions, the responses are likely additive rather than sequential. Further, as these responses appear to occur simultaneously, the data also infer that mucus shedding is not required for increases in CBF due to the presence of *V. fischeri*. Here, we report a range of changes in CBF (specifically, increases of 17%–26%) in response to various colonization processes and cues, which is comparable to the biologically relevant changes in CBF reported for other systems such as mouse nasal and tracheal epithelia (Jiao et al., 2011) and human nasal epithelia (Morse et al., 2001; Tratnjek et al., 2020).

The data presented here also bring up the question of where *V. fischeri* cells are being sensed and signaling important changes to the host in these initial minutes following hatching. The low number of *V. fischeri* cells, the quick response, and the mathematical modeling of the system suggest that the early sensing of these cells, while potentially at the light-organ ciliated fields, may occur elsewhere on the host’s body. As cephalopods have strong neural networks in their epithelial surfaces (Messenger, 2001), it is possible that the animal has “antennae” for the detection of *V. fischeri* cells either across the body surface or in specific, localized regions other than the light organ. The determination of mechanisms underlying this phenomenon offers a rich landscape for future research on this system. A principal approach will rely on the ability to delete and/or modify the expression of genes in *V. fischeri*, which will allow us to manipulate the activities of specific candidate proteins.

The results of this study are a “game-changer” to our view of the initial signaling during the process of host colonization in the squid-vibrio model, i.e., the demonstration of two periods of host-symbiont dialogue before the colonization event. Before the observations presented here, the major focus was upon the host’s initial sensing of and interaction with *V. fischeri* cells that occur with the formation of aggregations on the light organ surface (for review, see Nyholm and McFall-Ngai, 2021). By 3-h, these groups average a total of ∼5 cells attaching to the cilia of a few host cells near the pores; studies of that time point, in which the transcriptomes of symbiotic and non-symbiotic animals were compared, showed robust differential host gene expression between the two conditions (Kremer et al., 2013). It was concluded that the signaling of the observed changes in expression over these early hours was required to foster specific selection of *V. fischeri* cells, and the subsequent attraction of the aggregated bacteria into the host pores. In light of the data presented here, we cannot discount the possibility that earlier signaling drives the onset of these molecular processes and their eventual outcomes, i.e., within minutes, the first step in the process may also influence host gene expression. Future studies of the molecular responses of the host cells at these early time points should provide insights into the earliest measurable interactions between host and symbiont. However, even if a molecular mechanism is difficult to discover, the data here present a two-step process of symbiont signaling that is critical to colonization: 1) interactions with planktonic *V. fischeri* cells in the minutes following hatching that result in an increase in CBF that fosters a more efficient symbiont recruitment; and, 2) the process of aggregation of cells on the light-organ surface, where selection of the symbiont occurs.

We have shown that once the symbionts have colonized, they signal the loss of the ciliated field that potentiates light-organ colonization (Nyholm and McFall-Ngai, 2021). Concomitant with this morphogenesis is the associated loss of mucus shedding and a decrease in CBF before the ciliated cells are lost. To date, the authors know of no other system in which the loss of ciliated fields is induced by bacterial interactions. In establishment of the microbiome with ciliated surfaces in mammals, the cilia persist and, as such, it is likely that, whatever program of response to a coevolved microbiota is in play, it will also be retained.

## Data Availability

The original contributions presented in the study are included in the article/supplementary material, further inquiries can be directed to the corresponding author.

## Abbreviations

ASW, artificial seawater; CBF, ciliary beat frequency; FOSW, filtered offshore seawater; OSW, offshore seawater; PGN, peptidoglycan.
